# Balance Disorders in Children and Adolescents

**DOI:** 10.3390/children9081145

**Published:** 2022-07-29

**Authors:** Luca Oscar Redaelli de Zinis, Cristiano Balzanelli

**Affiliations:** 1 Department of Medical and Surgical Specialties, Radiological Sciences, and Public Health, Section of Audiology, University of Brescia, 25100 Brescia, Italy; 2 Pediatric Otolaryngology Head Neck Surgery Department, Children Hospital, ASST Spedali Civili, 25100 Brescia, Italy; 3 Vertigo Center—San Bernardino Polyclinic of Salò, 25087 Salò, Italy; balzanelli.cristiano@gmail.com

The prevalence of balance disorders in children and adolescents is extremely variable. Figures between 0.4% and 15% have been reported [1]. Fancello and coworkers [1] reviewed the literature of the last decade about the prevalence and the different etiologies with regards to age groups. They performed a selection using PRISMA guidelines and included 7 papers in their analysis. The total number of patients in the studies was 2470, there were subjects with multiple diagnosis with a total number of 3399. The age of the patients varied from 9 months to 21 years old, while a male to female ratio of 1:1.3. Vestibular migraine and other migraine variants represented the most frequent diagnosis (32.7%), followed by audio-vestibular disorders (23.9%), psychogenic vertigo (11.3%), neurological diseases (10.4%), post-traumatic vertigo (8.8%), motion sickness (3.6%), cardiovascular diseases (2.1%), and ophthalmic disorders (1.5%). Sporadic causes included dental, metabolic disorders and other systemic diseases representing 4.7% of diagnoses. The etiology was unknown in 0.9% of patients. The diagnoses in three age groups vase reported by 3 among the 7 papers: preschool (0–5 years old), elementary school (6–11 years old) and adolescents (12–18 years old). The results of the different age groups are reported in Figure 1. The migraine group contains patients from all the age groups (Figure 1). In the audio-vestibular group, benign paroxysmal positional vertigo accounted for 49% of disorders. Neurological disorders were particularly frequent in preschool children (Figure 1). Among neurological disorders, dysautonomia represented 46% of diagnoses.

The paper of Balzanelli et al. [2] reported new data on epidemiology of balance disorders in a total number of 472 subjects under the age of 18 in a 10-year period. Mean age was 11 ± 3 years and females represented 60% of patients. A detailed present and past history and a careful clinical and instrumental examination including study of eye movements of spontaneous, position, positioning, and evocative nystagmus along with the study of vestibulo-spinal reflexes was performed. If clinical vestibular evaluation was negative, rotatory chair test was added in the first years of the study and then substituted by video head impulse test. Audiological examination was always performed. Only when the evaluation was inconclusive, neuropsychiatric, ophthalmological, cardiological, or dental evaluation were scheduled. The rigorous application of this diagnostic work-up consented to obtain the definite diagnosis in 98.9% of patients. Vestibular loss (26.1%) was the most frequent diagnosis, followed by vestibular migraine (21.2%) and benign paroxysmal positional vertigo (10.2%). Benign paroxysmal vertigo of children was present in 8.5% cases (especially in females and in children under the age of 7). Psychogenic vertigo was found in 8.5% of their sample, ophthalmological disorders in 4.9%, dental disorders in 4.4%, hemodynamic orthostatic disorders in 4.0%, otitis media in 3.2%, epileptic disorders in 2.5%, postural disorders in 2.3%, trauma in 1.5%, Meniere’s Disease in 0.8%, and other neurological diseases in 0.6%.

A cross-sectional analysis to investigate the visually evoked postural responses by means of posturography in children with vestibular migraine when exposed to full-field horizontal optokinetic stimulation comparing to children with primary headache and controls was performed by Nocini et al. [3]. Static posturography was performed by a standardized stabilometric force platform. Statokinesigrams were obtained with open eyes, with closed eyes, and twice during a full-field horizontal optokinetic stimulation with electronystagmography to record optokinetic reflex parameters. The vestibular migraine and the control group were composed of 20 children, the primary headache group of 19. The groups were homogeneous in age and sex. The mean duration of illness and the time interval between the last headache or vertigo attack and the day of examination were similar in diseased groups. Statokinesigram with open eyes were similar in all groups, whereas Statokinesigram was significantly increased in both diseased groups. Stabilometric Romberg quotient was significantly increased in vestibular migraine subjects with respect to healthy subjects but not to primary headache subjects. Horizontal optokinetic stimulation destabilizing index was greater in vestibular migraine subjects compared to the other groups. Angular slow phase velocity, number of saccades, mean peak velocity of saccades induced by horizontal optic flow, and recorded by electronystagmogram were reduced in the group of vestibular migraine with respect to both the other groups.

In the paper of Bauer et al. [4] dynamic balance and shoulder mobility/stability of adolescents with borderline intellectual functioning was assessed through the Lower Quarter Y Balance Test (the patient stand on one leg while reaching out anterior, posteromedial and posterolateral directions with the other lower extremity) and the Upper Quarter Y Balance Test (the patient stand on one arm while reaching out medial, inferolateral, and superolateral directions with the other arm) and compared to age- and sex-matched controls. Normalized maximal reach distances were analyzed and compared between the groups. Significantly lower values of adolescents with borderline intellectual functioning were observed in both tests. The results confirm the hypothesis that intellectual functioning influences motor skills, suggesting the necessity to develop specific programs and to increase sport participation of young people with borderline intellectual functioning.

Viola and coworkers [5] analyzed the literature to summarize existing evidence related to pharmacological treatment vertigo in children. After a detailed description of sign and symptoms associated and of the causes of vertigo, they analyzed the therapies proposed for the most frequent balance disorders in children. Most of the studies concern migraine spectrum vertigo. The authors [5] suggest prophylaxis when attacks are more than three per month or if symptoms are severe. Unfortunately, randomized controlled clinical trials are rare and include only adults: there are only few studies of subjects under the age of 18 with several drugs including antiepileptics, antidepressants, antihistamines. No vestibular suppressant was efficacious in acute attacks whereas antiemetics were used for persisting symptoms. Vestibular neuritis is not common in children; its etiology is not defined, and in children it has a rapid evolution. Anti-nausea and antiemesis drugs can be used to alleviate symptoms. Benign paroxysmal positional vertigo is usually secondary to head trauma in children and pharmacological treatment is contraindicated. Children are rarely affected by Meniere’s disease in the ages between 8 and 10 years. As in adults, pharmacotherapy is debated: different drugs such as diuretics, anti-cholinergic, vestibular depressants, dihydroergotamine, ibudilast, trimetazidine, isosorbide dinitrate, antagonist on the H1, flunarizine, oral corticosteroids and nutrients such as Gingko Biloba have been proposed. Persistent postural-perceptual dizziness is a balance disorder that was recently described and consists of a functional neurological disorder associated with non-vertiginous dizziness and a sense of imbalance exacerbated by different situations. Diagnosis and treatment in children are not well-established; cognitive behavioral therapy or biofeedback therapy, physical therapy, associated with selective serotonin reuptake inhibitor or serotonin noradrenaline reuptake inhibitor have been proposed, but the use of antidepressant in children should be considered with caution. Motion sickness was the last balance disorder taken into consideration due to its increasing frequency. Different drugs including, scopolamine, antihistamines (dimenhydrinate, meclizine, promethazine, cinnarizine, cyproheptadine), ondasteton, and griffonia simplicifolia/magnesium have been studied for children. The conclusion of the authors [5] was that only few drugs are commonly used for balance disorders in children and adolescents and most of drugs are still used for research purposes.

In summary, this Special Issue covers epidemiological, diagnostic, and therapeutic aspects of balance disorders in children. I think that pediatricians can obtain useful information for their daily practice from this Special Issue, and researchers can be prompted to further studies on balance disorders in children and adolescents.

## Figures and Tables

**Figure 1:**
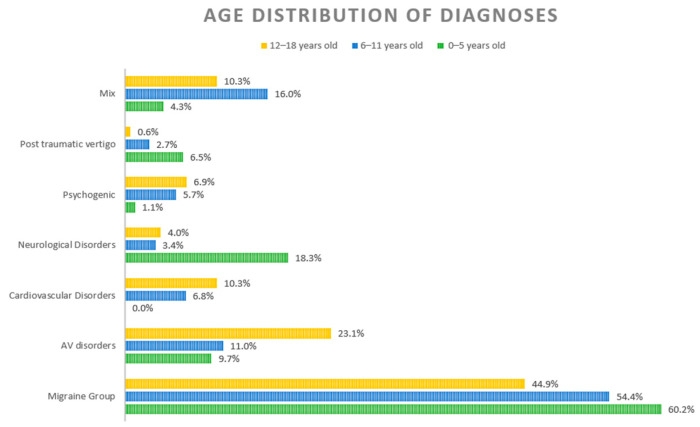
Distribution of different diagnoses according to age. (Reprinted with permission from Fancello et al. [1]).
